# Assessment of Oral Health-Related Quality of Life Using the Oral Impact on Daily Performance (OIDP) Instrument Among Secondary School Teachers of Modinagar, Uttar Pradesh: A Cross-Sectional Study

**DOI:** 10.7759/cureus.46256

**Published:** 2023-09-30

**Authors:** Monika Kumari, Basavaraj Patthi, Ashish Singla, Hina Naim Abdul, Manawar Ahmad Mansoor, Ananthalekshmy Rajeev

**Affiliations:** 1 Department of Public Health Dentistry, DJ College of Dental Sciences and Research, Modinagar, IND; 2 Department of Public Health Dentistry, Rajasthan Dental College, Jaipur, IND; 3 Department of Prosthetic Dental Sciences, College of Dentistry, Jazan University, Jazan, SAU

**Keywords:** children, school teachers, quality of life, practice, oral health

## Abstract

Introduction

Oral health has an impact on a person's general health, well-being, and quality of life. Due to their expertise and interactions with people, school staff members can actively contribute to the promotion of children's health if provided with the proper training.

Methodology

A cross-sectional survey was conducted to assess the "oral health-related quality of life" using the Oral Impact on Daily Performance (OIDP) instrument among the secondary school teachers of Modinagar, Uttar Pradesh. The study included a sample of 400 government secondary school teachers and 400 private secondary school teachers. The independent sample t-test was performed to assess the relationship between the mean of OIDP dimensions and the prevalence of dental caries. Any p-value less than 0.05 was considered significant. The data was analyzed using the SPSS version 20.0 software package (IBM Corp., Armonk, NY).

Results

In the present study, most of the participants (174 (43.5%) government teachers and 197 (49.2%) private teachers) were in the age group of 41-50 years. According to the Decayed, Missing, and Filled Teeth (DMFT) index, the mean decayed teeth present for government and private school teachers were 0.45 ± 0.503 and 0.41 ± 0.493, respectively, and the mean DMF scores for government and private teachers were 1.27 ± 0.736 and 1.03 ± 0.757, respectively. There was a positive correlation between the DMFT scores and the mean scores for the dimensions of the OIDP among both government and private secondary school teachers.

Conclusion

The poor dental health status of teachers' has a bad impact on everyday performance and academic work. The findings of the study highlight the need for oral health education and good oral health maintenance among school teachers since they are the ones who can easily influence the behavior of the children.

## Introduction

Health is a broad concept, with many different definitions. It has traditionally been described from a pathogenic, biomedical perspective as the absence of disease (Boorse, 1977) [1]. The World Health Organisation (WHO) first defined health as "a state of complete physical, mental, and social well-being, not merely the absence of disease or infirmity, and the capacity to lead a socially and economically productive life" in 1948 [2]. Over the years, there have been noticeable modifications to the idea of oral health. For a very long time, perspectives on oral health have been driven by physical elements of the mouth such as the absence of disease (National Library of Medicine, 1965) [3]. By viewing the mouth as a purely biological structure, the influence of mental and social factors is disregarded.

The emphasis on oral health has increasingly transitioned from a biologically defined disease notion to a multidimensional holistic perspective that includes physical, mental, and social factors (Gift & Atchison (1995); Locker (1997)). Oral health has an impact on general health, happiness, and quality of life. In addition to having a fatal outcome, oral diseases can have a negative impact on a variety of aspects of life, including eating, laughing, speaking, looking good, and even interpersonal relationships [4-6]. Oral diseases, such as dental caries or periodontal disease, are highly prevalent and their consequences can be physical, economic, social, or even psychological. The relationship between health and disease always has theoretical and practical importance. Likewise, oral problems and their treatment have their own importance in dental treatment as well as dental research [7-10].

Pathological conditions are not always the primary cause or origin of a disease or poor health. Mental health, quality of life, socioeconomic status, etc. also play an important role in the health of a person. Oral diseases badly impair the quality of life of individuals [11-13]. Health, disease, and quality of life are not points on a continuum but independent dimensions of human experience. Even though they are related, it is not necessary that they co-exist; they may be even experienced separately [14-16].

Disease as measured by professionals is philosophically and empirically different from disease and health as assessed by ordinary people themselves. Therefore, it is important to research the relationships between an individual's oral health status as determined by dental experts, their personal opinion of their oral health, and their oral health-related quality of life (OHRQoL). All the instruments used to measure OHRQoL have been verified globally and have been shown to be useful in terms of the number of dimensions [17-19]. Although these tools have been used to assess the quality of life related to oral health in many subpopulations, there are very little data regarding their application to certain workforces such as teachers who are involved in the promotion of school health [20,21]. Dental professionals play an important role in inspiring and guiding patients to adopt and maintain health habits that promote oral health, prevent disease, and facilitate successful treatment in case of disease. For encouraging individuals to adopt a healthy lifestyle, it is important for dentists to capture individuals' perspectives of oral health and OHRQoL first [22-24].

In a developing country like India with a population of more than 1 billion, where more than 70% of the people reside in rural areas, schools can function as a bridge between the seekers and the providers of oral health information [25,26]. Studies that have investigated oral health awareness among school children have revealed that they have a low level of knowledge. It is suggested that health education programs in schools be led by appropriately trained teachers. The role of a teacher is to not only give formal education to the children but also to mold their lives and make them better human beings. School teachers, with their expertise and contact with children, can actively contribute to student health promotion if they obtain adequate training and education [27,28]. Knowledge of oral diseases, and more significantly, the fact that most diseases are avoidable to a large extent, is a primary vehicle for improving children's oral health. School teachers have long been regarded as potentially crucial primary agents of socialization, capable of shaping schoolchildren's future, knowledge, attitude, and behavior [29]. According to a study conducted by Lawal FB et al. (2014), teachers play an essential part in developing children as effective role models to transfer values of life in and out of the classroom [30].

Although many studies have been conducted to assess OHRQoL, no study of school teachers has used the OIDP instrument for this purpose. Therefore, the present study was conducted to assess oral health-related quality of life using the OIDP instrument among secondary school teachers in Modinagar, India.

## Materials and methods

A descriptive cross-sectional study was performed in Modinagar using the Oral Impact on Daily Performance (OIDP) tool to examine the oral health-related quality of life among secondary school teachers. The Institutional Review Board of D.J. College of Dental Sciences and Research granted ethical clearance (DJD/IEC/2018/A-28). After thoroughly explaining the study's goal and methods, all of the school's administrators gave their official consent. Following a thorough explanation of the study's objectives and procedures, the study participants also provided written informed consent.

For the study's purposes, Modinagar was divided arbitrarily into four administrative zones, namely, East, West, North, and South. There were around 24 private and 36 government secondary schools in Modinagar city according to the documentary evidence of the District Education Office, Ghaziabad. From each zone, a total of 15 schools were selected randomly, thus making a total of 60 schools (15 schools x 4 zones = 60). From each zone of government and private schools, 100 teachers from government schools and 100 teachers from private schools were selected randomly to make a total sample size of 800 teachers (Government Secondary School Teachers: 400 + Private Secondary School Teachers: 400 = 800).

Inclusion criteria

All secondary school teachers working in Modinagar who were willing to participate in the study and teachers in the schools who gave their consent to take part in the survey were included.

Exclusion criteria

Specially and differently abled teachers, such as those on the autism spectrum, with early-stage dementia, etc., and teachers who were undergoing medical or dental treatment that could interfere with the examination's progress were excluded.

Sample size estimation

Based on the findings of the pilot study, the sample size for government and private secondary school teachers was established at 383.84 for the prevalence of dental caries (49%), 95% confidence level, and 5% acceptable error. After taking into consideration the expected attrition rate, the sample size for the government and private secondary school teachers was rounded off to 400 subjects in each group.

Scheduling of the survey

The study was systematically scheduled to spread over four months, from February to June 2019, among the secondary school teachers of Modinagar, India. A detailed monthly schedule was prepared well in advance by informing and obtaining consent from the principals of the schools. Based on the pilot study, the average time for the interview and clinical examination of each subject was calculated to be 10 to 15 minutes. The daily and weekly schedules were prepared accordingly. In a single day, a maximum of 40 subjects were examined for each school. The examination was carried out four days a week.

Data collection

A structured pretested proforma was used for data collection for the present study. The data collection comprised two sets of information: 1. Assessment of socio-demographic information and the oral health status of the study subjects based on the clinical examination according to procedures specified in the WHO Basic Oral Health Survey 2013 proforma; 2. The OIDP index was used to assess OHRQoL among secondary school teachers.

Clinical examination

The clinical assessment was carried out in the sequence/order prescribed in the WHO Basic Oral Health Survey Performa, with the sterilized instruments and materials checked by a single, trained, and calibrated examiner in accordance with the methods prescribed by WHO Basic Oral Health Survey Methodology 2013. A type III examination using a mouth mirror and probe was carried out for each subject. Clinical findings of the secondary school teachers were reported to the teachers at the end of the day of the examination. Reference slips were forwarded to the teachers, for information and necessary action. Teachers requiring treatment were referred to the Department of Public Health Dentistry, Divya Jyoti College of Dental Sciences & Research, Modinagar.

Recording of the OIDP questionnaire

Data were collected by means of an interviewer-administered questionnaire comprising two sections in the local language (Hindi). The first part was used to collect information on socio-demographic data, and the second part contained the OIDP scale. The OIDP index, a composite indicator that assesses the impacts of oral conditions on basic activities and behaviors of daily life, was chosen to assess OHRQoL that attributes impacts to specific oral conditions, thus facilitating its use in needs assessment for the different conditions. This scale contains a list of all oral problems that children are likely to perceive and includes an open answer for any unexpected, perceived problem. Teachers were asked to identify oral problems that they perceived in the last three months. Thereafter, teachers were individually interviewed, irrespective of their answers at the first step, to assess oral impacts on daily life in relation to eight daily performances.

The OIDP index assesses the serious oral impacts on eight daily performances: eating; speaking; cleaning teeth; relaxing, including sleeping; smiling, laughing, and showing teeth without embarrassment; maintaining emotional situations; studying, including going to school and doing homework; and having contact with other people.

For the OIDP, the overall impact score was the sum of all eight performance scores (ranging from 0 to 72) multiplied by 100 and divided by 72. Then, the prevalence of oral impacts was calculated as the percentage of children with respective overall scores higher than 0. An alternative method of reporting the severity of oral impacts, from the same data set, is to use the “intensity” and “extent” of impacts. The intensity refers to the most severe impacts on any of the eight performances or the highest performance score. It is classified into six levels: none, very little, little, moderate, severe, and very severe.

Statistical analysis

The data was analyzed using the SPSS v20.0 software package (IBM Corp., Armonk, NY). Descriptive statistics, such as mean, standard deviation, and percentage, were used. An Independent sample t-test was used to measure the association between the mean of OIDP dimensions and the prevalence of dental caries. The correlation between DMFT and mean OIDP scores among secondary school teachers was analyzed using Pearson's correlation. Any p-value less than 0.05 was considered significant.

## Results

A total of 400 government and 400 private school teachers were included. In the present study, most of the participants (174 (43.5%) government teachers, and 197 (49.2%) private teachers) were in the age group of 41-50 years. There were 190 (47.5%) male and 210 (52.5%) female government teachers, whereas private teachers included 184 (46%) males and 216 (54%) females. According to educational status, the present study revealed that 224 (56%) of government teachers and 236 (59%) of private teachers were post-graduates.

Among the 800 secondary school teachers, 189 (47.2%) government teachers and 173 (43.2%) private teachers had visited a dentist at least once. Most of the government teachers (339; 84.8%) and private teachers (331; 82.8%) in the current study used a toothbrush and toothpaste as oral hygiene methods. Regarding the frequency of tooth brushing, most secondary school teachers brushed only once daily. The demographic data of the study participants are shown in Table 1.

**Table 1:** Demographic data of the study participants

Demographic Data		Government		Private	
		N	%	N	%
Age	21-30 years	61	15.2%	61	15.2%
	31-40 years	94	23.5%	87	21.8%
	41-50 years	174	43.5%	197	49.2%
	51-60 years	71	17.8%	55	13.8%
Gender	Male	190	47.5%	184	46.0%
	Female	210	52.5%	216	54.0%
Educational status	Graduate	224	56%	236	59.0%
	Postgraduate	176	44%	164	41.0%
Smoking status	Smoker	13	3.2%	8	2.0%
	Non-smoker	387	96.8%	392	98.0%
Dental visits	Yes	189	47.2%	173	43.2%
	No	211	52.8%	227	56.8%
Dietary habits	Vegetarian	217	54.2%	207	51.8%
	Mixed	183	45.8%	193	48.2%
Oral hygiene method	Toothbrush and toothpaste	339	84.8%	331	82.8%
	Toothbrush and toothpowder	61	15.2%	69	17.2%
	Only finger	0	0%	0	0%
Duration of changing brush	0-3 months	148	37%	104	26.0%
	4-6 months	94	23.5%	246	61.5%
	7 months - 1 year	86	21.5%	41	10.2%
	More than 1 year	11	2.8%	0	0%
	When the bristles flare	61	15.2%	9	2.2%
Method of cleaning the teeth	Horizontal	230	57.5%	236	59.0%
	Vertical	168	42.0%	140	35.0%
	Circular	2	0.5%	24	6.0%
Frequency of tooth brushing	Once	236	59.0%	243	60.8%
	Twice	164	41.0%	157	39.2%
Time of cleaning the tooth	Before food	221	55.2%	227	56.8%
	After food	179	44.8%	173	43.2%
Use of interdental cleansing aids	Yes	139	34.8%	147	36.8%
	No	261	65.2%	253	63.2%

According to the DMFT index, the mean decayed teeth present for government and private school teachers were 0.45 ± 0.503 and 0.41 ± 0.493, respectively, and the mean DMF scores for government and private teachers were 1.27 ± 0.736 and 1.03 ± 0.757, respectively. The mean DMFT score was significantly higher among the government school teachers as compared to the private school teachers, which was found to be statistically significant (Table 2).

**Table 2:** Mean DMFT among secondary school teachers A P-value less than 0.05 was considered statistically significant. DMFT: Decayed, Missing, and Filled Teeth

	Location	Mean±SD	P-value
Decayed	Government	0.45±0.503	0.287
	Private	0.41±0.493	
Missing	Government	0.40±0.492	0.001^**^
	Private	0.21±0.408	
Filled	Government	0.41±0.493	0.943
	Private	0.41±0.492	
DMFT	Government	1.27±0.736	0.001^**^
	Private	1.03±0.757	

The overall mean OIDP scores for government and private teachers were 5.78 ± 8.52 and 4.76 ± 8.01, respectively. The difference between the government and private school teachers was found to be statistically significant for the dimension of eating only (p<0.05) (Table 3).

**Table 3:** Mean OIDP scores among secondary school teachers P≤0.05 is considered statistically significant. OIDP: Oral Impact on Daily Performance

OIDP Variables	Government	Private	Total	P-value
Eating	1.44 ± 1.40	1.36 ± 1.39	1.40 ± 1.39	0.419
Speaking	0.27 ± 0.84	0.20 ± 0.75	0.24 ± 0.80	0.217
Cleaning	1.22 ± 1.40	0.82 ± 1.27	1.02 ± 1.35	0.001^**^
Sleeping	0.34 ± 0.85	0.24 ± 0.78	0.29 ± 0.82	0.079
Smiling	0.46 ± 1.06	0.35 ± 1.00	0.40 ± 1.03	0.142
Emotion	1.44 ± 1.36	1.31 ± 1.33	1.37 ± 1.34	0.199
Work	0.18 ± 0.62	0.14 ± 0.59	0.16 ± 0.61	0.386
SocialContact	0.43 ± 0.99	0.34 ± 0.90	0.38 ± 0.95	0.170
Overall	5.78+8.52	4.76 ± 8.01	5.26 ± 8.29	0.637

The Pearson correlation of DMFT with OIDP variables was found to be statistically significant for eating, speaking, cleaning, sleeping, smiling, emotion, work, and social contact (p < 0.05) (Table 4).

**Table 4:** Pearson correlation between DMFT and mean OIDP scores among secondary school teachers P≤0.05 is considered statistically significant. OIDP: Oral Impact on Daily Performance; DMFT: Decayed, Missing, and Filled Teeth

OIDP Variables	Correlation Coefficient	P Value
Eating	0.73	0.001^**^
Speaking	0.29	0.001^**^
Cleaning	0.07	0.043^**^
Sleeping	0.16	0.001^**^
Smiling	0.68	0.001^**^
Emotion	0.83	0.001^**^
Work	0.48	0.001^**^
SocialContact	0.73	0.001^**^

## Discussion

Oral health knowledge is considered an essential prerequisite for health-related practices, and studies have shown that there is an association between increased knowledge and better oral health [2]. Teachers are an important and influential part of society and play an important role in the formation and modification of children's behaviors. Therefore, their knowledge and good oral health-related quality of life are essential for both their own and their children’s better oral health [25]. The lack of organized oral health programs in schools may be the reason for a lack of oral health knowledge among teachers in nations with lower incomes. As a result, it becomes necessary to evaluate their OHRQoL, which describes how an individual perceives their oral health and how it affects their everyday activities [9,10]. Hence, this present study focused on the OHRQoL among secondary school teachers in Modinagar, India.

The present study comprised school teachers from 38 government and private schools. Most of the teachers were in the age group of 41-50 years in both government (174; 43.5%) and private (197; 49.2%) schools. In a study by Amith HV et al. [26], 54% of the teachers were found to be above the age group of 45 years, while in a study by Ahmad et al. [27], only 12% of the teachers were reported to be above the age group of 40 years. In the present study, there were high proportions of female teachers in both government (210; 52.5%) and private schools (216; 54%) and these findings were in agreement with the study by Lawal et al. (2015) [28]. The present study showed that most of the teachers in both government (224; 56%) and private (236; 59%) schools were graduates as compared to postgraduates, and the results were in agreement with the study by Simon A et al. (2016) [29].

In the study, 189 (47.2%) of government teachers and 173 (43.2%) of private teachers had visited a dentist at least once. This was in accordance with the studies by Amith et al. [26] and Simon et al. [29], where 88% and 84.6% of the teachers, respectively, had visited a dentist earlier for a dental treatment to relieve pain. This indicated that the utilization of dental services was mainly for pain relief rather than prevention, and this may be due to the lack of knowledge among the teachers regarding the importance of routine dental check-ups.

The present study also revealed that 339 (84.8%) of government teachers and 331 (82.8%) of private teachers were using toothbrushes and toothpaste as oral hygiene methods. The results were in agreement with the study conducted by Simon et al. [29]. Regarding the frequency of tooth brushing, the study revealed that most of the government teachers (236; 59%) and private teachers (243; 60.8%) brushed only once daily and the findings are not in accordance with the studies by Ahmad et al. [27], Simon et al. [29], and Amith et al. [26] in which only 27.1%, 18.1% and 9.0% of the teachers, respectively, brushed only once daily.

According to the DMFT index, the mean DMFT score was observed to be significantly higher among the government school teachers (1.27±0.736) as compared to the private (1.03 ± 0.757) school teachers (p=0.001). In a study by Lawal FB et al., the mean DMFT score was found to be 0.61±1.60 [28]. The present study shows the highest mean of the OIDP variable among secondary school teachers for ‘eating’ (1.40) and the lowest mean was for ‘work’ (0.16). The findings were in agreement with the study by Saxena et al. in which the highest mean was for ‘eating’ (0.65) but the lowest mean was for ‘social contact’(0.06) [30]. In the study, DMFT and mean OIDP scores among secondary school teachers showed a significant correlation for all the variables of the OIDP (p≤0.05). These findings were similar to the results of the study conducted by Saxena et al. [30]. However, in the study conducted by Lawal et al., the association between DMFT and OIDP variables was found to have a non-significant association (p =0.053) [28].

The main disadvantage of the current study was its cross-sectional design, which complicated hypothesis testing because data on risk factors and outcomes were evaluated concurrently. Furthermore, the present study included a sample size of 800 individuals (400 government and 400 private), which can affect the generalizability of results. In order to comprehend and interpret OHRQoL measures among secondary school teachers, longitudinal studies are therefore required. Another limitation could be the self-reported bias of the OIDP, since the common person's judgment may differ from the clinician's point of view. Expansion of the oral disease prevention workforce and earlier disease-course interventions are required for optimal disease management.

By saving the time and expertise of dental professionals to handle complicated disorders, using primary healthcare workers for oral disease prevention and early detection will promote patient and family health and maximize the value of the primary healthcare workforce. The best place to give patients the information and self-care instructions, they may need to lower their risks for oral illness is in primary care settings. Therefore, the ideal choice for training schoolteachers about oral health would be primary health care professionals.

Recommendations

The study findings implicate the necessity of implementing a comprehensive oral health care program. Regular oral health education and promotion workshops for school teachers should be organized. All the programs should include free or low-cost routine dental treatment, fluoride administration in vehicles other than water, where water fluoridation is impractical, and health education with an emphasis on hygiene, diet, and nutrition. The seminars should emphasize the significance of dental health and its close connection to general health in order to change people's perceptions that oral health is unrelated to general health. Public-private partnerships and active involvement of the school teachers are needed for an organized approach to solving their oral health-related problems. The report may be submitted to the Ministry of Family and Health Welfare, the ministry should be engaged in mutual discussions as part of advocacy and lobbying.

However, the need for dental care was evident in all age groups examined in the study population. Governments at the federal and state levels must implement specific health policies that place a stronger emphasis on oral health promotion and prevention than on traditional curative care. It seems apparent that oral health problems cannot be resolved if the delivery of oral health care is provided by a dentist alone. Thus, the inclusion of primary health workers would play an important role in the delivery of preventive services. A national summit should be organized to bring researchers from all health professions together to examine the evidence further and plan strategic efforts to deal with the health and oral health of school teachers, as they play an important role in community development.

## Conclusions

The oral health status of school teachers significantly impacted their daily performance and school work when they experienced pain. Painless, but highly prevalent oral lesions, were not perceived to influence the teachers’ QoL. The prevalence of oral impact was high, with eating food being the most frequently affected performance. The highest impact was on ‘eating’ and the lowest on ‘speaking and pronouncing’ in the study population, which in turn, had an impact on their QoL. The present study results indicate that the OIDP inventory is applicable for use in descriptive questionnaire surveys among secondary school teachers of Modinagar. There are various oral conditions that contribute significantly to the incidence of impacts, namely, toothache and missing teeth. Although the prevalence of impacts was high, the impact score was not, as many individuals had their quality of life affected at low levels. This reveals a need for further longitudinal studies to better understand and interpret OHRQoL measures in such populations. It was able to distinguish fairly reliably between subjects with and without clinically defined oral disorders. Significant associations were found between the OIDP items on the one hand, and the global self-reported oral health indicator on the other, providing further evidence of the applicability of this instrument.

Regarding the oral health quality of life of secondary school teachers, the findings of the study highlight the need for oral health education and good oral health maintenance since teachers can easily influence the behavior of the children. Dental colleges, in collaboration with other institutes, can take the initiative to formulate health education programs for the teachers by various means such as health talks, lectures, demonstrations, and training to help create awareness regarding oral health maintenance and identify various oral health problems.
